# Daily Household Electricity Consumption in Community-Dwelling Older Individuals With Cognitive Impairment: Prospective Cohort Study

**DOI:** 10.2196/71265

**Published:** 2025-10-16

**Authors:** Yuki Nakagawa, Shigeo Tanabe, Hikaru Kondo, Koki Tan, Soichiro Koyama, Shin Kitamura, Akiko Kada, Takuma Ishihara, Takuaki Yamamoto, Junya Denda, Hideaki Kimata, Taisuke Yamanaka, Ryosuke Umezawa, Yoshinobu Nakahashi, Yohei Otaka

**Affiliations:** 1 Department of Rehabilitation Medicine, School of Medicine, Fujita Health University, 1-98 Dengakugakubo, Kutsukake, Toyoake, 470-1101, Japan, +81-562-93-2167; 2 Graduate School of Health Sciences, Fujita Health University, Toyoake, Japan; 3 Faculty of Rehabilitation, School of Health Sciences, Fujita Health University, Toyoake, Japan; 4 Research Center for Robotic Smart Home and Activity Assistive Technology, Fujita Health University, Toyoake, Japan; 5 Department of Rehabilitation, Fujita Health University Hospital, Toyoake, Japan; 6 Center for Translational Research, Fujita Health University, Toyoake, Japan; 7 Innovative and Clinical Research Promotion Center, Gifu University, Gifu, Japan; 8 Chubu Electric Power Co Inc, Nagoya, Japan; 9 Necolico LLC, Chiyoda-ku, Japan; 10 Japan Data Science Consortium Co. Ltd., Bunkyo-ku, Japan

**Keywords:** detection, digital biomarker, nonwearable device, prevention, screening, sensor, smart meter, thermal sensitivity

## Abstract

**Background:**

Various digital biomarkers have been explored to detect cognitive impairment in community-dwelling older individuals, among which electricity consumption (EC) data obtained from smart meters are novel and promising because they pose no burden to the individuals.

**Objective:**

The study aimed to explore the potential of EC as a digital biomarker to screen older individuals with cognitive impairment living alone.

**Methods:**

We recruited 40 older individuals living alone and recorded their 1-year daily household EC data. We used the Japanese version of the Montreal Cognitive Assessment to categorize participants into 2 groups: those with and without cognitive impairment. As the pattern of daily household EC is different between lower and higher temperature ranges because of the use of heating and cooling equipment, we divided the daily household EC into 3 temperature ranges. Using a linear mixed model, we evaluated the association between daily household EC, daily outside temperature, and the groups.

**Results:**

After excluding 12 participants, they were categorized into 2 groups: those with (10/28, 36%) and without cognitive impairment (18/28, 64%). The daily household EC data consisting of 9391 points showed two characteristics: (1) daily household EC was significantly lower in the group with cognitive impairment than in the group without cognitive impairment in the high temperature range (2.158 kWh at 25 °C, *P*=.02; 3.712 kWh at 30 °C, *P*<.001). The increase in EC with rising temperature from 25 °C to 30 °C was less in the group with cognitive impairment (2.387 kWh, *P*<.001) than in the group without cognitive impairment (3.940 kWh, *P*<.001); and (2) a tendency for lower daily household EC in the group with cognitive impairment was observed in the moderate temperature range (1.795 kWh at 15 °C, *P*=.06; 1.582 kWh at 20 °C, *P*=.08).

**Conclusions:**

The group with cognitive impairment may use less cooling equipment in the high temperature range and fewer home appliances in the moderate temperature range. Daily household EC might be useful in screening cognitive impairment in older individuals living alone.

## Introduction

Mild cognitive impairment (MCI) is an early stage of cognitive decline, often involving memory loss or other cognitive deficits, while individuals retain the ability to perform most activities of daily living (ADL) independently [1]. Although MCI is associated with a high risk of dementia, individuals with MCI may return to normal cognitive function [1], with a reversion rate of approximately 18% (95% CI 14%‐22%) [2]. In addition, interventions targeting lifestyle and ADL at the MCI stage may delay or prevent further cognitive decline [3]. Therefore, early detection of MCI and subsequent early intervention are important [4]. However, most patients with cognitive decline visit a hospital after their symptoms have substantially progressed, and only a few patients visit a hospital while they are at the MCI stage [5].

Various researchers have attempted to develop assessment methods that allow for the screening of community-dwelling older individuals in their own homes. Recently, readily available digital biomarkers, such as smartwatch-based step counters and smart ring–based continuous heart rate monitors, have attracted growing interest in cognitive assessment, which takes advantage of widely available mobile and wearable technologies [6]. However, older individuals require more support than younger individuals when using digital technology. Specifically, older individuals need simpler and more user-friendly mobile devices, such as fewer buttons, larger font sizes, and better color contrast. In this context, digital biomarkers, which can be obtained from sensor devices installed in homes, are considered promising for detecting MCI as they work without user interaction [6].

Electricity consumption (EC) data obtained using smart meters have garnered attention as novel digital biomarkers [7]. EC data are advantageous because they do not require user interaction and can be obtained while monitored individuals are living naturally at home. Numerous countries have been promoting their installations in households, with an estimated 729.13 million installations worldwide as of 2019 [8].

In a previous study, the association between the lifestyles and behaviors of older individuals with cognitive impairment was explored using household EC data obtained from an additional specialized electricity sensor with high precision and high sampling frequency [9]. Specifically, the ADL were estimated by identifying the amount of time each home appliance was used [9]. The use of common smart meters to assess behaviors requires no additional cost and imposes no burden on device management or invasion of privacy. Because patients with MCI perform various characteristic instrumental ADL (IADL) [10], the early detection of MCI using ordinary EC data obtained from common smart meters may be feasible.

For the early detection of MCI, it is important to focus on older individuals living alone as the risk of developing dementia is increased in these individuals (relative risks=1.30, 95% CI 1.15‐1.46) [11]. Screening for cognitive function in older individuals living alone is useful because their families are unlikely to notice their cognitive decline.

In this study, we aimed to explore the possibility of using ordinary EC data as a digital biomarker to screen older individuals with cognitive impairment living alone.

## Methods

### Study Design and Participant Recruitment

This was an exploratory prospective cohort study with a convenience sample. Data were obtained between April 1, 2022, and March 31, 2023. Study participants were recruited from Toyoake City in the Aichi prefecture, located in the center of the Japanese archipelago (at a latitude of 35° north) on September 9, 2021. The participants were recruited from local senior centers, city halls, and other community-based locations. The inclusion criteria were as follows: (1) aged ≥65 years, (2) living alone and independently, (3) never diagnosed with dementia, (4) users of smart meters, and (5) consent to provide EC data. The exclusion criteria were as follows: (1) individuals using electric water heaters that caused characteristic EC patterns and (2) individuals whose EC data were determined to be inappropriate for analysis based on visual inspection and researcher consensus. The latter criterion was applied post hoc to exclude data with clear anomalies or artifacts (eg, persistent signal dropout or unusually large use patterns) that could not be reasonably attributed to physiological or behavioral factors. The process was carefully documented and applied consistently across all participants.

### Ethical Considerations

This study was approved by the Research Ethics Committee of Fujita Health University (HM23-108) and reported in accordance with the Strengthening the Reporting of Observational Studies in Epidemiology (STROBE) guidelines (Checklist 1). All participants provided written informed consent before enrollment. Participants were informed that their data would be used exclusively for research purposes. To ensure privacy and confidentiality, all collected data were anonymized before analysis and stored in encrypted formats on a secure computer, accessible only to authorized study personnel. No personally identifiable information is included in the manuscript, and individual participants cannot be identified from any images, text, or supplementary materials. Participants did not receive any monetary compensation; however, they were provided with feedback on their health screening results.

### Smart Meters

**Figure 1.**
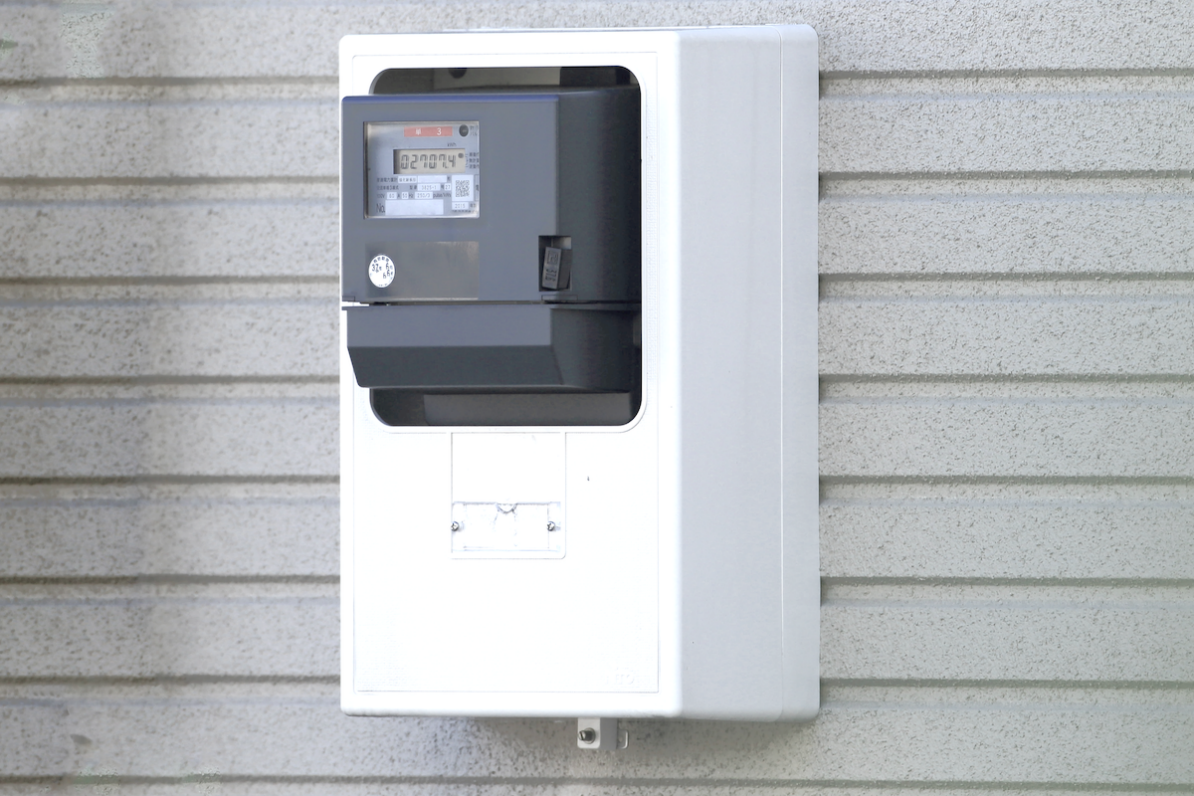
Example of a smart meter commonly used in Japan to collect household electricity consumption data. This figure shows a typical smart meter, usually installed outside Japanese residences. In this study, 30-minute interval electricity consumption data in watt-hours were obtained through these meters using a home energy management system, a home gateway, and the wireless smart utility network. This image is for illustrative purposes only and does not depict the actual device used in the study.

Smart meters are devices that measure EC in real time and automatically transmit information to electricity companies or individual households in a digital format [8]. The common smart meters used in Japan automatically transmit information data on household EC (the total amount of electricity used) every 30 minutes and are typically installed outside homes (Figure 1). According to a survey by the Ministry of Economy, Trade, and Industry of Japan, smart meters were installed in approximately 85% (69.17 million units) of households in Japan by March 2021 [12]. For a general purpose, data on household EC are transmitted to electricity companies and used to calculate electricity charges and understand the overall energy demand in the region. In addition, each household can connect a home energy management system (HEMS) device, which is a home energy monitoring system, to a smart meter to receive relevant data at any given point. In this study, we used 30-minute interval EC data obtained directly from common smart meters via the wireless smart utility network and internet with HEMS and a built-in home gateway.

### Data Collection

The basic sociodemographic data, cognitive function, and physical function of the study participants were assessed by 3 health care professionals (physical therapists) at the time of participation in the study. The collection and assessment items included age, sex assigned at birth, residence information, Japanese version of the Montreal Cognitive Assessment (MoCA-J) score [13], Frailty Screening Index [14], Kihon Checklist [15], walking speed [16], and grip strength [17].

The MoCA-J is a Japanese version of the Montreal Cognitive Assessment (MoCA) that is used to evaluate cognitive function [13]. The MoCA is used to detect MCI in older individuals [18]. MoCA-J scores range from 0 to 30, with lower scores indicating cognitive decline. Participants with a MoCA-J score ≤25 were classified as the group with cognitive impairment, whereas those with a score ≥26 were classified as the group without cognitive impairment [13]. Groups with and without cognitive impairment were the primary comparison variables in this study. The MoCA-J is more sensitive than the Mini-Mental State Examination for detecting MCI in older individuals (sensitivity 93%; specificity 89%) [13]. This test has been widely used in numerous large-scale studies on cognitive function. Furthermore, the reliability and validity of the MoCA in detecting MCI have been well established in Japanese populations compared to conventional cognitive tests [13].

The Frailty Screening Index is used to test frailty and consists of 5 self-report questionnaires [14]. If 3 or more questions are answered “yes,” the patient may have frailty and is at increased risk of death or need for nursing care [14]. The Kihon Checklist is widely used in Japan to identify older individuals at risk for requiring support or nursing care [15]. A score of ≥8 out of 25 items indicates frailty [15].

Walking speed is an indicator of lower limb muscle strength. Older individuals with a slower walking speed (<0.6 m/s) have a higher risk of future hospitalization and deterioration of health status than those with a faster walking speed (≥0.6 m/s) [16]. Similarly, grip strength is an indicator of upper limb muscle strength and overall physical function. It is used as a cutoff value for sarcopenia in Asians, defined as the ability to grip <28 kg for men and <18 kg for women [17].

The time-stamped 30-minute values of household EC were obtained from a smart meter. Daily household EC was calculated using 30-minute values×48 per day obtained between April 1, 2022, and March 31, 2023. For data on daily household EC that were completely or partially missing during the study period, the 1-day data for the corresponding participant were excluded from the analysis.

Outside temperature data were obtained from the Japan Meteorological Agency website [19]. The daily outside temperature was the mean of the hourly values observed from 12 AM to 12 PM. Data from Obu City, a city neighboring Toyoake City, were used because the mean temperature of Toyoake City was unavailable. The city hall distance between the experimental site and the outside temperature observation site was approximately 8 km, and the elevation difference was <2 m.

### Statistical Analyses

Data on participant demographics, cognitive function, and physical function were analyzed using descriptive statistics. The characteristics of the participants with and without cognitive impairment were compared using the Fisher exact test for categorical variables and the Mann-Whitney *U* test for continuous variables. Annual trends in daily household EC and their association with daily outside temperature were presented in scatter plots or lines.

Daily individual household EC data were repeatedly measured for 1 year. Therefore, a linear mixed model introducing a random intercept was used to analyze the daily household EC. The normality of the residuals was verified using histograms. The linear mixed model included daily outside temperature, study group, and the interaction between daily outside temperature and group as fixed effects and participants as random effects. To account for nonlinearity, a cubic spline was considered for temperature. Based on previous findings [20], 25 °C, the boundary between high and medium temperatures, and 15 °C, the boundary between medium and low temperatures, were set as knots for the spline. Significant interaction indicated different daily household EC and daily outside temperature associations between the groups. Based on the predicted values obtained from the model, a contrast analysis was performed on the difference in the daily household EC between groups for each temperature. Because the model includes nonlinear effects of temperature, interaction terms, and main effects, we evaluated the differences between groups by comparing the predicted values at specific temperatures rather than performing statistical tests on individual coefficients. Furthermore, the difference for every 5 °C change in daily household EC was calculated for each group. As the mechanism of missing power consumption data can be assumed to be missing at random, we considered estimation without bias possible using a linear mixed model. Analyses were performed using R (version 4.3.1; R Foundation for Statistical Computing) and the *lme4* package. The 2-sided significance level was set at *P*<.05. Since this study was exploratory, no adjustments were made for multiple comparisons.

## Results

### Participants

**Figure 2.**
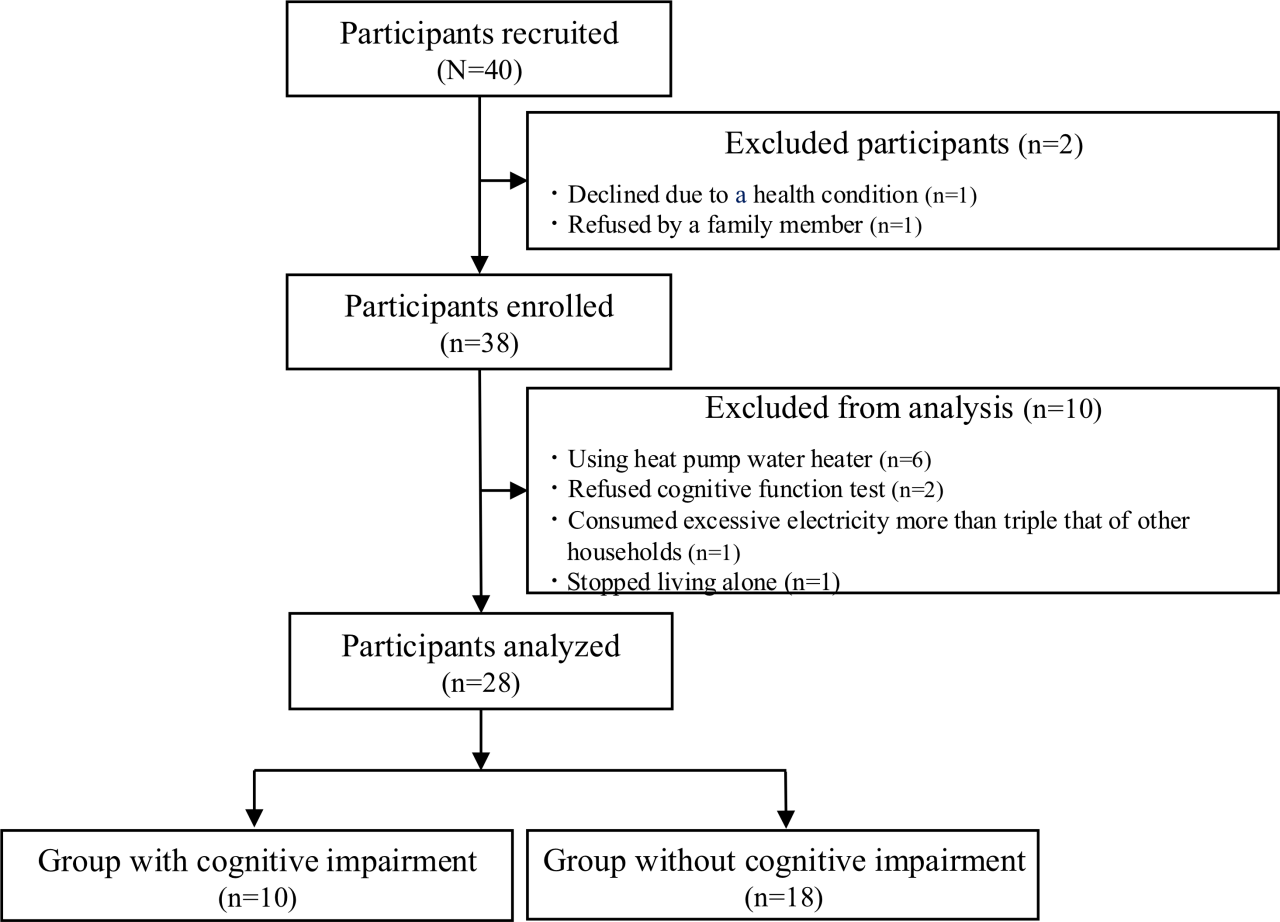
Flow diagram of inclusion and exclusion criteria for participants among community-dwelling older individuals in Toyoake City, Japan (April 2022-March 2023): an exploratory prospective cohort study. This figure illustrates the screening process, inclusion and exclusion criteria, and final sample size for a prospective cohort study investigating the association between cognitive impairment and household electricity consumption (EC) in community-dwelling older individuals in Toyoake City, Japan.

The participant flow diagram is shown in Figure 2. A detailed breakdown of the reasons for exclusion is provided in this diagram. A total of 40 individuals met the initial inclusion criteria. After excluding 12 participants, 28 were enrolled in the study. Based on the MoCA-J scores, 10 (36%) participants were categorized as the group with cognitive impairment and 18 (64%) participants were categorized as the group without cognitive impairment.

### Participant Characteristics

Table 1 shows the participant demographics and the results of cognitive and physical function tests. A significant difference in MoCA-J scores was observed between the two groups (*P*<.001). However, no significant differences in age, sex assigned at birth, residence information, Frailty Screening Index, Kihon Checklist, walking speed, or grip strength were observed between the 2 groups.

**Table 1.** Sociodemographic and clinical characteristics of the participants in Toyoake City, Japan (April 2022-March 2023).

Variable	Group with cognitive impairment (n=10)	Group without cognitive impairment (n=18)	*P* value
Age (y), median (IQR)	77 (76‐83)	79 (74‐86)	.76
Sex, n (%)			.43
Male	3 (30)	4 (22.2)	
Female	7 (70)	14 (77.8)	
Type of residence, n (%)			.38
House	6 (60)	15 (83.3)	
Apartment	4 (40)	3 (16.7)	
MoCA-J^a^ (score), median (IQR)	21.5 (20‐24.5)	28 (27‐29)	<.001
Frailty screening index (score), median (IQR)	1 (0‐2)	0 (0‐1)	.10
Kihon checklist (score), median (IQR)	2 (1–6)	3 (2–5)	.54
Gait speed (m/s), median (IQR)	1.2 (1.1‐1.4)	1.3 (1.0‐1.4)	.75
Grip strength (kg), median (IQR)	24.1 (17.8‐34.9)	26.6 (21.7‐30.1)	.95
Male	36.5 (35.4‐40.3)	34.6 (26.1‐38.0)	.63
Female	17.8 (17.5‐24.8)	24.8 (21.5‐27.8)	.13

a MoCA-J: Japanese version of the Montreal Cognitive Assessment.

### Data Points

A total of 10,220 data points were collected from 28 participants over 365 days. Of these, 515 (5%) data points were missing owing to the data extraction schedule, and 314 (3.1%) data points were missing owing to poor connections between the wireless local area networks, HEMS devices, and smart meters. Consequently, there were 9391 (91.9%) available data points.

### Daily Household EC and Daily Outside Temperature

**Figure 3.**
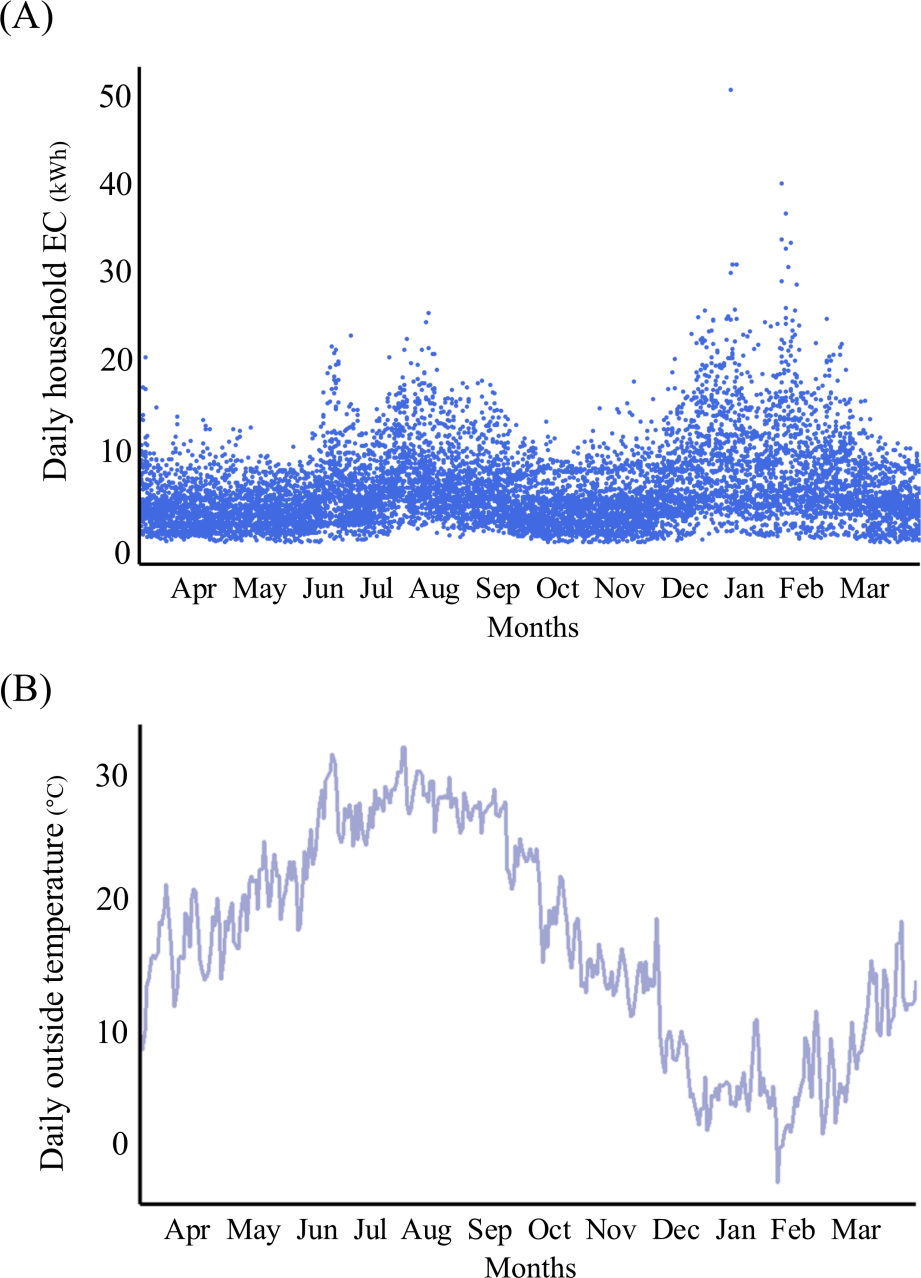
Daily household electricity consumption (EC) data and outside temperature for the year (Apr [April] 2022-Mar [March] 2023). (**A**) Daily household EC data collected from 28 community-dwelling older individuals in Toyoake City, Japan. The horizontal axis represents the month, and the vertical axis shows household EC in kilowatt-hours (kWh). (**B**) Daily outside temperature data in Obu City, Japan, which is adjacent to Toyoake City. The horizontal axis represents the month, and the vertical axis shows the daily outside temperature (°C). The lines represent the daily outside temperature for each day. Aug: August; Dec: December; Feb: February; Jan: January; Jul: July; Jun: June; Nov: November; Oct: October; Sep: September.

**Figure 4.**
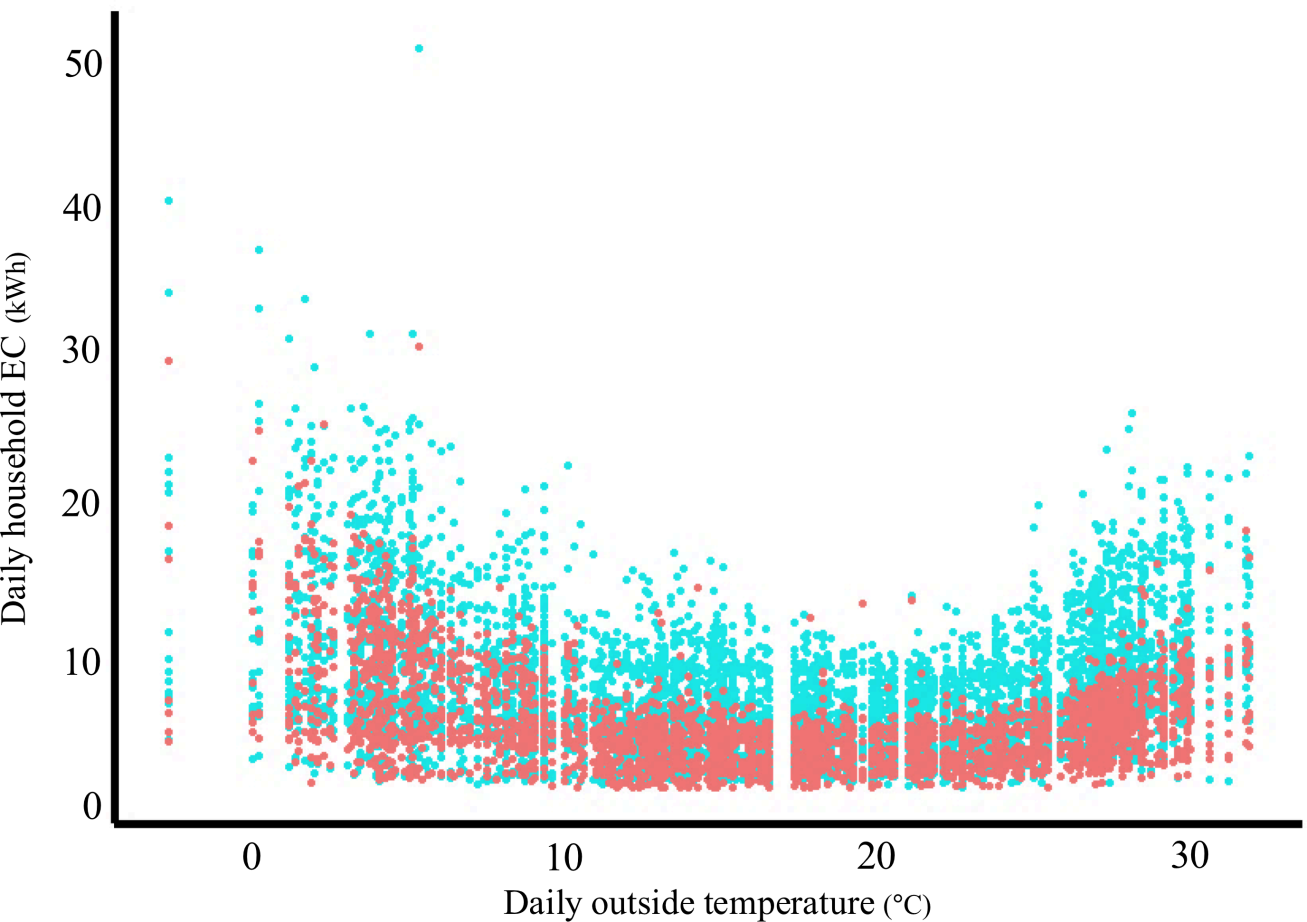
Scatter plot showing the association between daily household electricity consumption (EC) and outside temperature (April 2022-March 2023). Each dot represents 1 observation from a total of 28 community-dwelling older individuals. The horizontal axis shows the daily outside temperature (°C) in Obu City, Japan, which is adjacent to Toyoake City, Japan, and the vertical axis indicates daily household EC in Toyoake City (kWh). Red and blue dots represent individuals with (n=10) and without (n=18) cognitive impairment, respectively.

Figures 3A and 3B show the changes in daily household EC for all study participants and the daily outside temperature of the city near the study location, respectively, from April 1, 2022, to March 31, 2023. The daily household EC tended to increase in both summer (July to September) and winter (December to February), while the daily outside temperature increased during summer and decreased in winter. The daily household EC ranged from 1.4 kWh to 51.1 kWh, and the daily outside temperature ranged from −1.1 °C to 32.2 °C during the study period. Figure 4 shows the association between daily outside temperature and daily household EC. In both low and high temperature ranges, the daily household EC increased as the temperature became colder or warmer. The daily household EC tended to be lower in the group with cognitive impairment than in the group without cognitive impairment.

### Association Between Daily Outside Temperature and the Groups on the Daily Household EC

**Figure 5.**
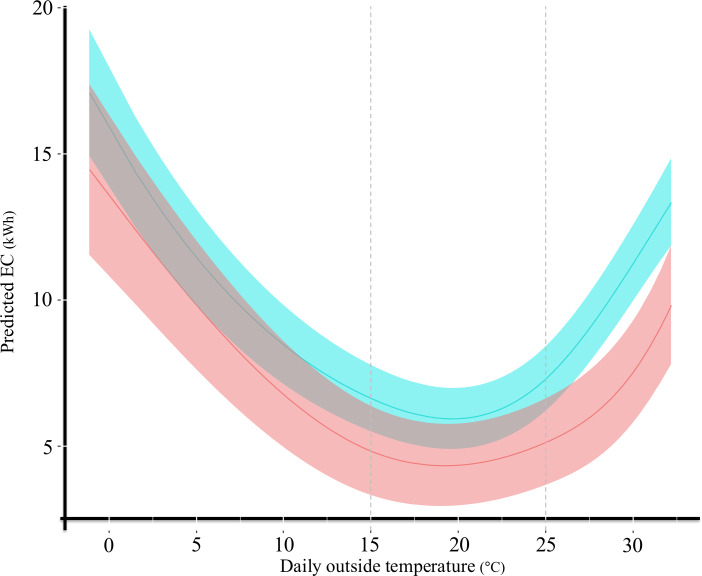
Electricity consumption (EC) lines predicted based on daily outside temperature in the 2 groups (April 2022-March 2023). The horizontal axis represents daily outside temperature in Obu City, Japan, which is adjacent to Toyoake City, Japan (°C), and the vertical axis shows the predicted daily household EC (kWh) based on a linear mixed model. Solid lines represent group-level predicted means, and shaded areas indicate 95% CIs. Red and blue lines represent the groups with (n=10) and without (n=18) cognitive impairment, respectively.

The type III test of ANOVA for the interaction terms was significant (*P*<.001), suggesting that the association between daily household EC and outside temperature may differ in shape between the groups. Multimedia Appendix 1 presents the detailed results of the type III ANOVA, including the main effects of temperature and group, and their interaction. Table 2 presents the results of the contrast analysis based on the linear mixed model, and Table 3 summarizes the estimated differences between groups for each 5 °C increase in temperature. The *P* values reported in these tables were unadjusted for multiplicity. Figure 5 illustrates the predicted lines obtained from the linear mixed models. Details of the estimated fixed effects, including spline terms, are provided in Multimedia Appendix 2.

**Table 2.** Estimated daily household electricity consumption (EC) at fixed outside temperatures (April 2022-March 2023) based on least-squares means from a linear mixed model.

Temperature (°C)^a^	Least-squares means for the group with cognitive impairment^b^ (kWh)	Least-squares means for the group without cognitive impairment^b^ (kWh)	Differences (kWh)	*P* value
0	13.576	15.884	2.308	.19
5	9.833	11.464	1.631	.25
10	6.771	8.466	1.696	.14
15	4.837	6.631	1.795	.06
20	4.352	5.934	1.582	.08
25	5.128	7.286	2.158	.02
30	7.515	11.226	3.712	<.001

a Outside temperatures in Obu City, Japan, which is adjacent to Toyoake City, Japan (°C).

b Least-square means indicates the estimated mean daily household EC (kWh).

**Table 3.** Estimated differences in daily household electricity consumption (EC) across adjacent temperature intervals (April 2022-March 2023) based on linear mixed model estimates.

Temperature^a^ interval (°C)	Difference^b^ (kWh; 95% CI)	*P* value
Group with cognitive impairment		
5‐0	−3.743 (−5.081 to −2.405)	<.001
10‐5	−3.063 (−3.923 to −2.202)	<.001
15‐10	−1.934 (−2.788 to −1.080)	<.001
20‐15	−0.484 (−1.344 to 0.375)	.64
25‐20	0.776 (−0.065 to 1.617)	.09
30‐25	2.387 (1.465 to 3.309)	<.001
Group without cognitive impairment		
5‐0	−4.420 (−5.380 to −3.460)	<.001
10‐5	−2.998 (−3.635 to −2.361)	<.001
15‐10	−1.835 (−2.468 to −1.202)	<.001
20‐15	−0.697 (−1.334 to −0.061)	.02
25‐20	1.352 (0.727 to 1.977)	<.001
30‐25	3.940 (3.262 to 4.619)	<.001

a Outside temperatures in Obu City, Japan, which is adjacent to Toyoake City, Japan.

b The difference represents the estimated mean difference in daily household EC obtained from community-dwelling older individuals in Toyoake City between the specified temperature intervals.

In the high temperature range, daily household EC was significantly lower in the group with cognitive impairment than in the group without cognitive impairment (2.158 kWh at 25 °C, *P*=.02; 3.712 kWh at 30 °C, *P*<.001). The increase in EC with rising temperature from 25 °C to 30 °C was less in the group with cognitive impairment (2.387 kWh; *P*<.001) than in the group without cognitive impairment (3.940 kWh; *P*<.001).

In the moderate temperature range, no significant differences were observed in daily household EC between the groups with changes in daily outside temperature. However, the group with cognitive impairment tended to show a lower daily household EC than the group without cognitive impairment at both 15 °C (1.795 kWh; *P*=.06) and 20 °C (1.582 kWh; *P*=.08).

In the low temperature range, no significant differences were observed in daily household EC between the groups with changes in outside temperature. However, a main effect of daily outside temperature was observed, indicating that daily household EC increased with decreasing temperature in both groups (group with cognitive impairment: 3.743 kWh from 0 °C to 5 °C, 3.063 kWh from 5 °C to 10 °C, 1.934 kWh from 10 °C to 15 °C, all *P*<.001; group without cognitive impairment: 4.420 kWh from 0 °C to 5 °C, 2.998 kWh from 5 °C to 10 °C, 1.835 kWh from 10 °C to 15 °C, all *P*<.001).

## Discussion

### Principal Findings

In this study, a combined analysis of daily household EC data obtained from common smart meters and daily outside temperature data suggests that there are differences in EC between individuals with and without cognitive impairment living alone. These results suggest that EC data could be useful as a digital biomarker for screening cognitive function in older individuals living alone.

### Comparison With Prior Studies

The daily household EC of both groups tended to increase with changes in the daily outside temperature in both low and high temperature ranges. These trends can be attributed to the use of heating and cooling equipment, respectively. Daily household EC increased when daily outside temperatures rose or fell beyond 13 °C to 25 °C in Shanghai, China, owing to the influence of heating and cooling equipment use [20]. Similarly, daily household EC increased when the highest outside temperature decreased or increased in the Kyushu region of Japan (latitude 30°‐33° north) [21].

In the high temperature range, the group with cognitive impairment used less electricity than the group without cognitive impairment in terms of the increase in daily household EC associated with the rise in outside temperature. This may be due to reduced sensitivity to the rise in outside temperature and the lack of appropriate indoor temperature control using cooling equipment. A mouse study indicated a delayed response latency to heat stimuli in mice with dementia compared to that in healthy mice [22]. In human studies, the threshold for temperature perception in older individuals with mild to moderate Alzheimer disease (AD) is higher than that in those without AD (median 34.0, IQR 33 °C-36 °C for older individuals with AD vs median 32.0, IQR 32 °C-34 °C for those without AD) [23]. Other previous studies have shown that older individuals with cognitive impairment have an increased risk of developing heat stroke [24,25]. Patients with psychiatric disorders, such as dementia, are at a higher risk of hospitalization than healthy individuals because of heat-related illnesses, including heat stroke [24]. The risk of hospitalization for patients with dementia increases by 4.5% (95% CI 2.9%‐6.1%) for every 1 °C increase in the daily mean temperature >17 °C [25]. Further research is needed to clarify whether reduced use of cooling equipment—potentially owing to a higher threshold for sensing elevated body temperature—in individuals with cognitive impairment contributes to their heightened vulnerability to heat stroke.

In the moderate temperature range, a trend of a lower daily household EC was observed in the group with cognitive impairment than in the group without cognitive impairment. This suggests that individuals in this group used fewer household appliances during the seasons when heating and cooling equipment are not typically used. IADL assessments have shown a decline in the ability of individuals with MCI to use household appliances compared to older individuals without cognitive impairment [26]. A previous study using an additional specialized electricity sensor with high precision and high sampling frequency has reported shorter use times for appliances such as induction heaters in spring for individuals with cognitive impairment [9].

In the low temperature range, the group with cognitive impairment showed a tendency toward lower daily household EC; however, no significant differences were observed between the two groups. This finding is inconsistent with those of a previous study, which demonstrated the effect of seasons on daily household EC, such as reduced use time of certain home appliances, including air conditioners, in winter among individuals with cognitive impairment [9]. Although the reason for this inconsistency remains unclear, it could be attributed to differences in individual sensitivity to cold temperatures. A previous study indicated that such sensitivities could vary widely, with variation in thermal sensation sensitivity reaching as high as 50% (ie, 1.5 times for the highest and 0.5 times for the lowest relative to the mean value) [27]. In addition, various findings on the impact of cognitive decline on sensitivity to low temperatures have also been reported, ranging from no difference to higher sensitivity compared with that of control groups [28,29]. For instance, a study involving mice indicated that the response latency of dementia model mice to cold stimuli (5 °C cold water) was equivalent to that of healthy mice [28]. In contrast, a study on humans found that individuals with mild to moderate AD had lower resistance to cold stimuli (immersing hands in water at 4 °C for 2 min) than individuals without cognitive impairment [29].

Although the reasons for the different characteristics between the low and high temperature ranges in this study are unclear, differences in neural pathways may have contributed. A study on the thermosensory neural network in mice revealed that the neural circuits responsible for identifying comfortable temperature environments differ according to their responses to heat and cold. Specifically, neuronal tracing identified 2 segregated groups of neurons in the lateral parabrachial nucleus (LPB): one projecting to the median preoptic nucleus (MnPO), a thermoregulatory center (LPB→MnPO neurons), and the other to the central amygdaloid nucleus (CeA), a limbic emotion center (LPB→CeA neurons). While LPB→MnPO neurons include separate subgroups activated by heat or cold exposure, LPB→CeA neurons were only activated by cold exposure [30].

### Interpretation of Model Results

Although the interaction term between group and temperature in the linear mixed model was statistically significant, the interpretation of this result requires caution. Our model included both linear and nonlinear spline terms for temperature and their interactions with group. However, the significance of the interaction term does not directly imply uniform group differences across all temperature values. Rather, it suggests that the shape of the association between temperature and daily EC may differ between groups. To address this complexity, we performed contrast analyses using model-derived predicted values to estimate group differences at specific temperature levels (eg, 0 °C, 5 °C, and 10 °C). This approach allowed us to determine where meaningful differences were most likely to occur while accounting for the full structure of the model. The results from this contrast analysis indicated clear group differences in the high temperature range, where individuals with cognitive impairment used significantly less electricity. We believe this analytic strategy improves interpretability and helps avoid complex conclusions based on several regression coefficients that include interactions and nonlinear terms.

### Limitations

As the number of participants in this study was small (N=28), a verification study with a larger sample size is required. The groups with and without cognitive impairment comprised 10 and 18 participants, respectively. However, the demographic characteristics and physical function of the study participants were generally similar between the two groups, except for the presence or absence of cognitive decline. This finding suggests that the study was not influenced by specific personal or environmental factors. Residential type (eg, house or apartment) may also influence EC. Although apartment residents tended to consume less electricity, the distribution of residence types was relatively balanced between the groups, suggesting that its impact on the group comparisons was likely minimal. Nonetheless, future studies should explicitly consider residential type as a potential confounding factor.

Validation of these findings in other regions with different latitudes and climatic conditions is necessary. This study was conducted in specific regions of Japan, which limits the generalizability of our findings. However, the results may also be applicable to areas with mid-latitude, as well as warm and humid climates, similar to Toyoake City, Aichi Prefecture (35° north latitude; eg, coastal areas of Korea and southern China). In addition, while the study participants were older individuals living alone and were not engaged in paid employment, which we assumed resulted in relatively comparable income levels, individual income could still influence energy consumption patterns. Previous research has shown that as income increases, households’ weather sensitivity remains the same for hotter days in the summer but increases during the winter [20]. In this study, we did not record the exact proportion of participants who had completed higher education. However, we acknowledge the potential for socioeconomic selection bias. Specifically, this may affect the generalizability of our findings to populations with different socioeconomic backgrounds. According to the Organisation for Economic Co-operation and Development [31], individuals with tertiary education earn nearly twice as much on average as those without upper secondary education across Organisation for Economic Co-operation and Development countries. Li et al [20] demonstrated that higher-income households exhibit greater sensitivity to temperature changes in winter, suggesting that EC patterns may vary according to socioeconomic status. Thus, our results may have been influenced by this socioeconomic selection bias. This factor was not explicitly controlled for in our study, and future research should examine the potential impact of household income on energy consumption behavior. Furthermore, this study focused primarily on daily outdoor temperature as the environmental factor influencing EC. Other environmental factors, such as variations in daylight hours, were not considered. Daylight duration may affect daily activity rhythms and, by extension, patterns of EC, particularly among older individuals with cognitive impairment. Prior research has demonstrated that changes in light exposure can influence rest-activity patterns, mood, and agitation levels in individuals with dementia [32].

### Conclusions

In summary, the use of cooling equipment in the high temperature range and various household appliances in the moderate temperature range might have influenced the differences in daily household EC between the groups with and without cognitive impairment. If changes in cognitive function can be longitudinally revealed using daily household EC, further advancements in research can enable the screening of cognitive function in older individuals living alone without the psychological and physical burdens associated with conventional screening of cognitive function. Reducing hospital visits for cognitive function assessment and monitoring of physical and cognitive conditions by health care professionals may contribute to the improvement of health management and quality of medical care for older individuals. In addition, these findings may lead to the development of new technologies to support ADL and IADL in older individuals.

This study revealed, for the first time, differences in daily household EC between individuals with and without cognitive impairment using only data obtained from common smart meters without additional specialized sensors. In addition, this study demonstrated the possibility of using daily household EC data with daily outside temperature as a digital biomarker for the early detection of cognitive decline in older individuals living alone. These findings may have major implications for health care management since early detection of cognitive decline can enable early intervention.

## Supplementary material

10.2196/71265 Multimedia Appendix 1 Type III ANOVA table for the linear mixed model.

10.2196/71265 Multimedia Appendix 2 Estimated values of fixed effects in a linear mixed model.

10.2196/71265 Checklist 1 STROBE checklist.

## Abbreviations

AD: Alzheimer disease
ADL: activities of daily living
CeA: central amygdaloid nucleus
EC: electricity consumption
HEMS: home energy management system
IADL: instrumental activities of daily living
LPB: lateral parabrachial nucleus
MCI: mild cognitive impairment
MnPO: median preoptic nucleus
MoCA: Montreal Cognitive Assessment
MoCA-J: Japanese version of the Montreal Cognitive Assessment
STROBE: Strengthening the Reporting of Observational Studies in Epidemiology
