# Supplementary material for: Daily Household Electricity Consumption in Community-Dwelling Older Individuals With Cognitive Impairment: Prospective Cohort Study
Source: JMIR Form Res. 2025 Oct 16;9:e71265. doi: 10.2196/71265 (PMC12530452; doi:10.2196/71265)
Supplement: Multimedia Appendix 1 [file formative-v9-e71265-s001.docx]

**Multimedia Appendix 1.** Type III ANOVA table for the linear mixed model.

The table shows results from a Type III Analysis of Variance (ANOVA), evaluating the effects of outside temperature, cognitive function group, and their interaction on daily household electricity consumption.

Group is a dummy variable indicating cognitive status:

0 = group without cognitive impairment

1 = group with cognitive impairment

The interaction term (eg, Temperature: Group) tests whether the relationship between temperature and electricity consumption differs by cognitive function group.

P-values are reported with three decimal places, including leading zeros (eg, .090). Statistical significance was set at α = 0.05.

|  | Mean Square | Degrees of Freedom | *F* | *P* |
| --- | --- | --- | --- | --- |
|  |  |  |  |  |
| Temperature | 5,194.630 | 5 | 1149.552 | < .001 |
| Group | 9.084 | 1 | 2.010 | .166 |
| Temperature: Group | 82.066 | 5 | 18.161 | < .001 |
