# Supplementary material for: Daily Household Electricity Consumption in Community-Dwelling Older Individuals With Cognitive Impairment: Prospective Cohort Study
Source: JMIR Form Res. 2025 Oct 16;9:e71265. doi: 10.2196/71265 (PMC12530452; doi:10.2196/71265)
Supplement: Multimedia Appendix 2 [file formative-v9-e71265-s002.docx]

**Multimedia Appendix 2.** Estimated values of fixed effects in a linear mixed model.

The table shows the estimated fixed effects from a linear mixed model examining daily household electricity consumption as the outcome variable. The model includes terms for outside temperature (modeled using polynomial and piecewise polynomial functions), cognitive function group, and their interaction effects.

Group is a dummy variable indicating cognitive status:

0 = group without cognitive impairment

1 = group with cognitive impairment

Variables Temperature, Temperature2, and Temperature3 represent the first- to third-order polynomial terms of outside temperature.

Variables [Temperature-15]3 and [Temperature-25]3 represent piecewise cubic polynomial terms with knots at 15 °C and 25 °C, respectively.

Interaction terms (eg, Temperature: Group) represent differences in temperature effects between cognitive groups.

The intercept represents the estimated electricity consumption at baseline conditions. P-values are reported with three decimal places, including leading zeros (eg, .090). Statistical significance was set at α = 0.05.

| Variable | *β* | 95% confidence interval | | *P* |
| --- | --- | --- | --- | --- |
|  |  | Lower | Upper |  |
|  |  |  |  |  |
| Intercept | 17.073 | 14.923 | 19.223 | < .001 |
| Temperature | −6.004 | −7.158 | −4.849 | < .001 |
| Temperature^2^ | −10.866 | −12.155 | −9.578 | < .001 |
| Temperature^3^ | −11.836 | −14.035 | −9.636 | < .001 |
| [Temperature-15]^3^ | −6.035 | −8.632 | −3.438 | < .001 |
| [Temperature-25]^3^ | −3.743 | −6.581 | −0.905 | .015 |
| Group | −2.626 | −6.243 | 0.990 | .166 |
| Temperature: Group | 1.739 | −0.261 | 3.739 | .090 |
| Temperature^2^: Group | 0.060 | −2.118 | 2.237 | .958 |
| Temperature^3^: Group | 1.821 | −1.887 | 5.529 | .345 |
| [Temperature-15]^3^: Group | −1.709 | −6.071 | 2.652 | .450 |
| [Temperature-25]^3^: Group | −0.890 | −5.662 | 3.882 | .718 |
